# The Settlement of Madagascar: What Dialects and Languages Can Tell Us

**DOI:** 10.1371/journal.pone.0030666

**Published:** 2012-02-21

**Authors:** Maurizio Serva

**Affiliations:** Dipartimento di Matematica, Università dell'Aquila, L'Aquila, Italy; Universita' del Piemonte Orientale, Italy

## Abstract

The dialects of Madagascar belong to the Greater Barito East group of the Austronesian family and it is widely accepted that the Island was colonized by Indonesian sailors after a maritime trek that probably took place around 650 CE. The language most closely related to Malagasy dialects is Maanyan, but Malay is also strongly related especially for navigation terms. Since the Maanyan Dayaks live along the Barito river in Kalimantan (Borneo) and they do not possess the necessary skill for long maritime navigation, they were probably brought as subordinates by Malay sailors. In a recent paper we compared 23 different Malagasy dialects in order to determine the time and the landing area of the first colonization. In this research we use new data and new methods to confirm that the landing took place on the south-east coast of the Island. Furthermore, we are able to state here that colonization probably consisted of a single founding event rather than multiple settlements.To reach our goal we find out the internal kinship relations among all the 23 Malagasy dialects and we also find out the relations of the 23 dialects to Malay and Maanyan. The method used is an automated version of the lexicostatistic approach. The data from Madagascar were collected by the author at the beginning of 2010 and consist of Swadesh lists of 200 items for 23 dialects covering all areas of the Island. The lists for Maanyan and Malay were obtained from a published dataset integrated with the author's interviews.

## Introduction

The Malagasy language (as well all its dialects) belongs to the Austronesian linguistic family. This was definitively established in [1] where a particularly close relationship is also shown between Malagasy and Maanyan, which is spoken by a Dayak community of Borneo. A relevant contribution also comes from loanwords of other Indonesian languages such as Ngaju Dayak, Buginese, Javanese and Malay [2], [3]. In particular, Malay is very well represented in the domain of navigation terms. A very small amount of vocabulary can be associated with non-Austronesian languages (for example Bantu languages for faunal names [4]).

The Indonesian colonizers reached Madagascar by a maritime trek at a time that we estimated in a recent paper [5] to be around 650 CE, a date which is within the widely accepted range [2], [3]. In the same paper we found a strong indication that the landing area was in the south-east of the Island. This was established assuming that the homeland is the area exhibiting the maximum of current linguistic diversity. Diversity was measured by comparing lexical and geographical distances.

In this paper we confirm the south-east location as the area of landing (where the population dispersal originated). Furthermore, we find out that colonization consisted in a single founding event. Therefore, it is unlikely that there were multiple settlements. Any eventual subsequent landings did not appreciably alter the linguistic equilibrium. Our study starts from the consideration that Maanyan speakers, who live along the rivers of Kalimantan, do not have the necessary skills for long-distance maritime navigation. The most reasonable explanation [2], [3] is that they were brought as subordinates by Malay sailors. For this reason we reexamine the internal kinship relations among all the 23 Malagasy dialects but we also perform a comparison of all these variants with both Malay and Maanyan. These new results concerning Malagasy dialects and their relations with the two Indonesian languages are examined with new methods which all confirm that the landing took place on the south-east coast of the Island.

The vocabulary used for the present study was collected by the author with the invaluable help of Joselinà Soafara Néré at the beginning of 2010. The dataset, which can be found in [6], consists of 200-words Swadesh lists [7] for 23 dialects of Malagasy from all the areas of the Island. The orthographical conventions are those of standard Malagasy. Most of the informants were able to write the words directly using these conventions, while a few of them benefited from the help of one or more fellow townsmen. For any dialect list two different speakers were interviewed. The complete list of speakers is provided by Table S1 of Supporting Information. Finally, the lists for Maanyan and Malay were obtained from a published dataset [8] integrated with the author's interviews.

The method that we use [9], [10] is based on an automated measure of distance between all pairs of languages obtained by comparison between their 200-words Swadesh lists.

## Results

### Malagasy dialects

The number of Malagasy dialects we consider is 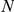 = 23; and the output of our method, when applied only to these variants, is a matrix with 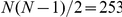 non-trivial entries representing all the possible lexical distances among dialects. This matrix is explicitly shown in Table S2 of Supporting Information.

**Figure 1:**
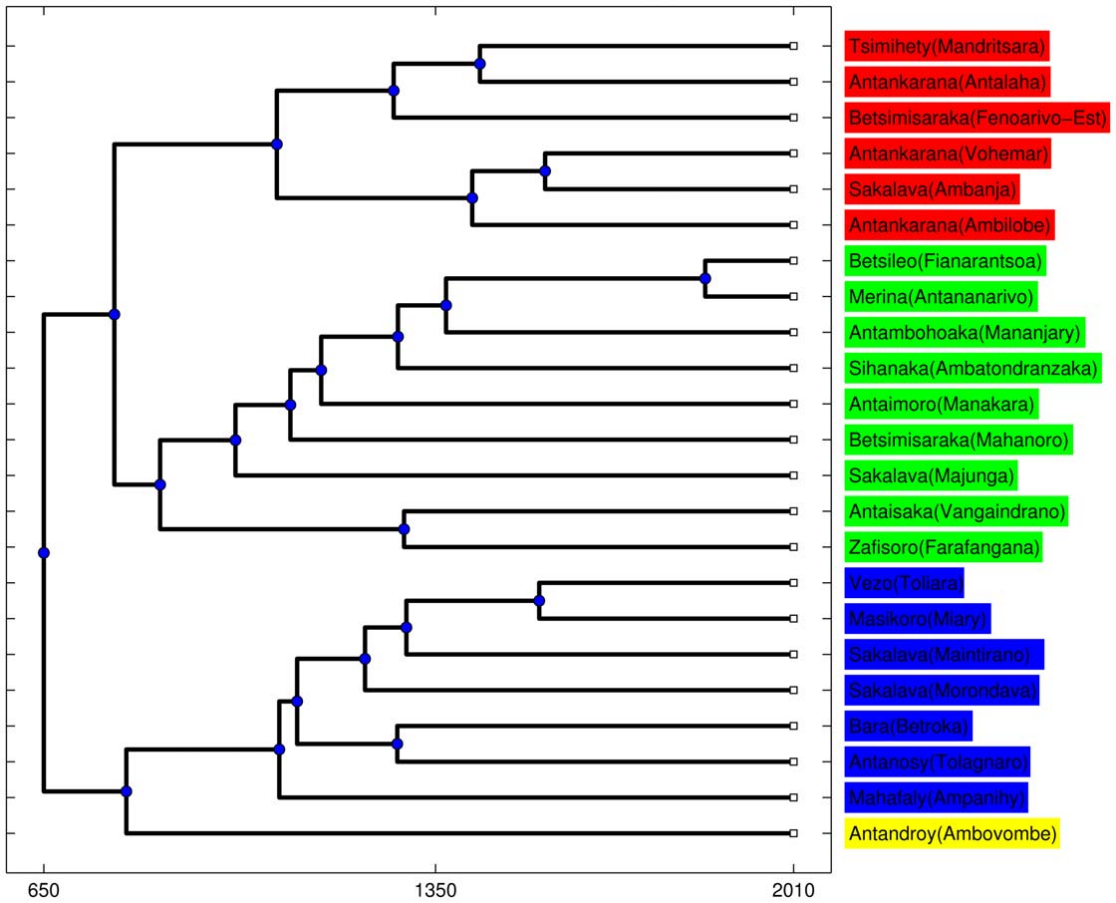
Phylogenetic tree of 23 Malagasy dialects realized by Unweighted Pair Group Method Average (UPGMA). In this figure the name of the dialect is followed by the name of the town where it was collected. The phylogenetic tree shows a main partition of the Malagasy dialects into four main groups associated with different colors. The strict correspondence of this cladogram with the geography can be appreciated by comparison with Fig. 2.

**Figure 2:**
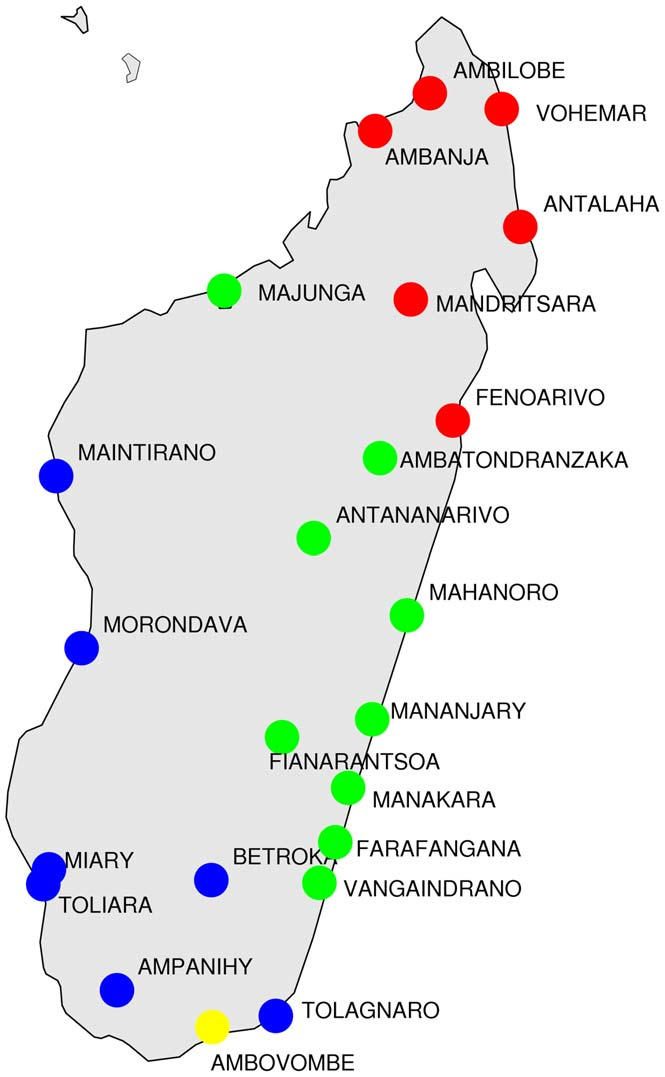
Geography of Malagasy dialects. The locations of the 23 dialects are indicated with the same colors as in Fig. 1. Each dialect is identified by the name of the town where it was collected.

The information concerning the vertical transmission of vocabulary from proto-Malagasy to the contemporary dialects can be extracted by a phylogenetic approach. There are various possible choices for the algorithm for the reconstruction of the family tree (see [5] for a discussion of this point), we show in Fig. 1 the output of the Unweighted Pair Group Method Average (UPGMA). In this figure the name of the dialect is followed by the name of the town where it was collected. The input data for the UPGMA tree are the pairwise separation times obtained from the lexical distances by means of a simple logarithmic rule ([9], [10]). The absolute time-scale is calibrated by the results of the SCA analysis, which indicate a separation date 650 CE [5]. The phylogenetic tree in Fig. 1 interestingly shows a main partition of Malagasy dialects into two main branches (east-center-north and south-west) at variance with previous studies which gave a different partitioning [11] (indeed, the results in [11] coincide with ours if a correct phylogeny is applied, see [5] for a discussion of this point.) Then each of the two branches splits, in turn, into two sub-branches whose leaves are associated with different colors. In order to demonstrate the strict correspondence of this cladogram with the geography, we display a map of Madagascar (Fig. 2) where the locations of the 23 dialects are indicated with the same colors of the leaves in Fig. 1. We note the relative isolation of the Antandroy variant (yellow).

**Figure 3:**
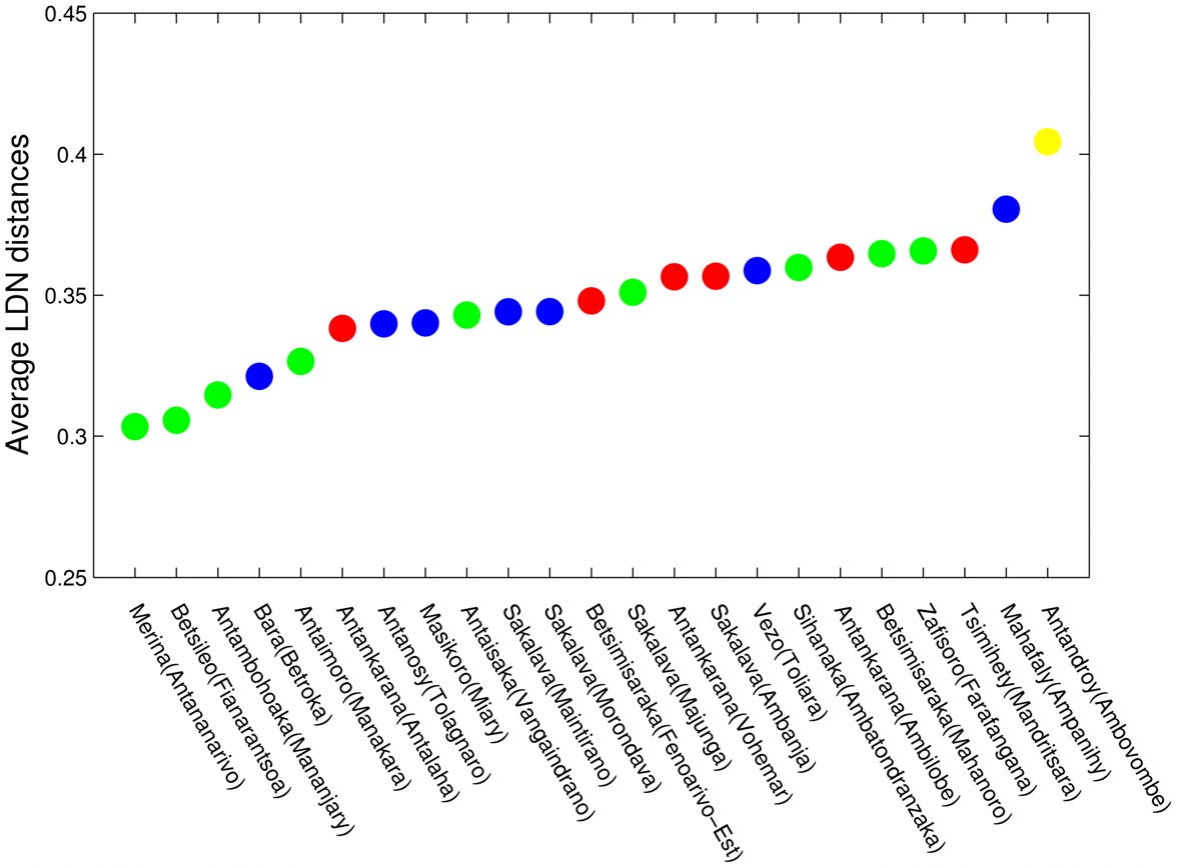
Average distance of the Malagasy dialects from all the others. The 23 dialects are colored as in Fig. 2. Highlands dialects (Antananarivo, Fianarantsoa and Betroka) together with south-east coast dialects (Mananjary and Manakara) show the smallest average distance.

Up to now, we only have shown the consistency of the approach, which can be appreciated by comparison between Fig. 1 and Fig. 2. We start our investigation by computing the average distance of each of the dialects from all the others (see Fig. 3). Antandroy has the largest average distance, confirming that it is the overall most deviant variant (something which is also commonly pointed out by other Malagasy speakers). We further note that the smallest average distance is for Merina (the official language), Betsileo and Bara, which are all spoken on the highlands. The fact that the Merina has the smallest average distance is possibly partially explained by the fact that this variant is the official one. However, as we will show later by means of a comparison of Malagasy dialects with Malay and Maanyan, this cannot be the only explanation. More interestingly we remark that the Antambohoaka and Antaimoro variants, which are spoken in Mananjary and Manakara, also have a very small average distance from the other dialects. Both of these dialects are spoken on the south-east coast of Madagascar in a relatively isolated position; therefore, this is the first evidence for the south-east as the homeland of the Malagasy language and, likely, as the location of the first settlement.

The identification of the southeastern coast of Madagascar as the landing area for the Indonesian colonizers is supported by geographical considerations. In fact, there is an Indian Ocean current which goes from Sumatra to Madagascar. When Mount Krakatoa erupted in 1883, pumice arrived on the south-east coast where the Mananjary River opens into the sea. Furthermore, during the Second World War, the same area saw the arrival of pieces of wreckage from ships sailing between Java and Sumatra that had been bombed by the Japanese air force. Notice that the mouth of the Mananjary River is where the town of Mananjary is presently located, and it is also close to Manakara. The Indonesian ancestors of today Malagasy probably profited from this current, which they possibly entered sailing through the Sunda strait.

### Dialects, Malay and Maanyan

The classification of Malagasy (and its dialects) among the Greater Barito East languages of Borneo as well as the particularly close relationship with Maanyan is beyond doubt. However, Malagasy also underwent influences from other Indonesian languages such as Ngaju Dayak, Javanese, Buginese and, particularly, from Malay which exhibits the most relevant relationship after Maanyan.

**Figure 4:**
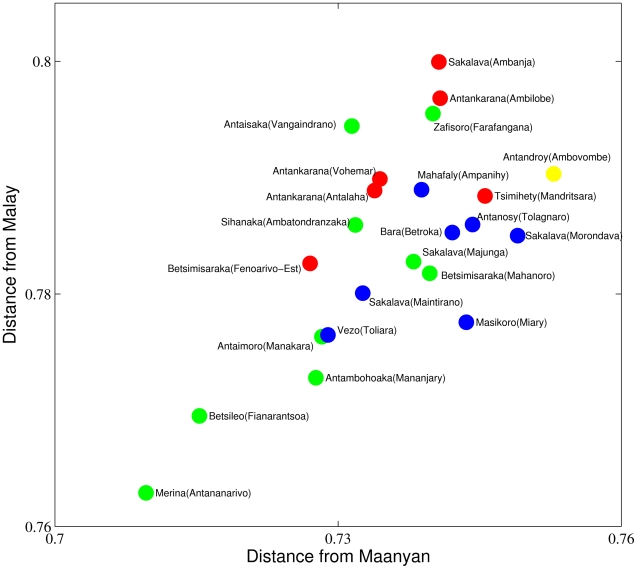
Lexical distances of Malagasy dialects from Malay and Maanyan. The 23 dialects are indicated with the same colors as in Fig. 2. Highlands dialects together with south-east coast Mananjary and Manakara dialects show the smallest distance from both of the two Indonesian languages.

If we consider the 23 dialects together with Malay and Maanyan, not only do we have to compute the 253 internal distances, but also we have to determine the 23×2 = 46 distances of each of the dialects from the two Indonesian languages. These new distances are displayed in Fig. 4.

First of all we observe, as expected, that the largest of the distances from Maanyan is smaller then the smallest of the distances from Malay. This simply reflects the fact that Malagasy is first of all an East Barito language. Then we also observe that Malagasy dialects seem to have almost the same relative composition. In fact, all the points in Fig. 4 have almost the same *distance from Malay*/*distance from Maanyan* ratio. This is a strong indication that the linguistic makeup is substantially the same for all the dialects and, therefore, that they all originated by the same founding population of which they reflect the initial composition. The conclusion is that the founding event was likely a single one and subsequent immigration did not significantly alter the linguistic composition.

Indeed, looking more carefully, one can detect a little less Malay in the north since the red circles have a larger ratio than all the others. This cannot a be a consequence of a larger African influence in the vocabulary due to the active trade with the continent and the Comoros Islands. In this case both the Maanyan and Malay component of the vocabulary would be affected. Instead, this may be the effect of Malay trading which, according to Adelaar [2], [3], continued for several centuries after colonization.

Noticeably, some dialects changed less from the proto-language (Antananarivo, Fianarantsoa, Manajary, Manakara), in fact, their distances both from Maanyan and Malay are smaller then those of the other dialects. This is probably the most relevant phenomenon, and we underline that the variants which are less distant on average with respect to the other dialects (Fig. 3) are also less distant with respect to Malay and Maanyan (Fig. 4). Therefore, the fact that Merina is closer to the other dialects cannot be explained merely by the fact that it is the official variant.

We have checked whether the picture which emerges from Fig. 4 is confirmed by comparing the Malagasy dialects with other related Indonesian languages. The result is positive, and in particular the dialects of Manajary, Manakara, Antananarivo and Fianarantsoa seem to be closer to most of the Indonesian languages which we compare them to. Note that Manajary and Manakara are both in the previously identified landing area on the south-east coast while Antananarivo and Fianarantsoa are in the central highlands of the Island. This suggests a scenario according to which there was a migration on the highlands of Madagascar (Betsileo and Imerina regions) shortly after the landing on the south-east coast (Manakara, Manajary).

In conclusion, both the average distances in Fig. 3 and the distances from related Indonesian languages point to the south-east coast as the area of the first settlement. This is also indicated by the fact that linguistic diversity is higher in that region (see [5]).

Finally, we remark that the Antandroy variant (Ambovombe) is the most distant from Maanyan and among the most distant dialects from Malay, showing it again to be the most deviant dialect. It is not clear whether its divergent evolution was due to internal factors or to specific language contacts which are still to be identified.

## Discussion

The main open problem concerning Malagasy is to determine the composition of the population which settled the Island. Adelaar writes: *Malay influence persisted for several centuries after the migration. But, except for this Malay influence, most influence on Malagasy from other Indonesian languages seems to be pre-migratory. (…) I also believe it possible that the early migrants from south-east Asia came not exclusively from the south-east Barito area, in fact, that south-east Barito speakers may not even have constituted a majority among these migrants, but rather formed a nuclear group which was later reinforced by south-east Asian migrants with a possibly different linguistic and cultural background (and, of course, by African migrants). Whatever view one may hold on how the early Malagasy were influenced by other Indonesians, it seems necessary that we at least develop a more cosmopolitan view on the Indonesian origins of the Malagasy. A south-east Barito origin is beyond dispute, but this is of course only one aspect of what Malagasy dialects and cultures reflect today. Later influences were manifold, and some of these influences, African as well as Indonesian, were so strong that they have molded the Malagasy language and culture in all its variety into something new, something for the analysis of which a south-east Barito origin has become a factor of little explanatory value*.

In order to clarify the problem raised by Adelaar, it is necessary to understand the Malagasy relationships with other Indonesian languages (and possibly African ones). The fact that the use of some words is limited to one or more dialects was already taken into account in previous studies. For example it is known that the word *alika* which refers to *dog* in Merina (the official variant) is replaced by the word *amboa* of African origin in most dialects. Nevertheless, the study of Malagasy dialects in comparison with Indonesian languages is a still largely unexplored field of research. Each dialect may provide pieces of information about the history the language, eventually allowing us to for track the various linguistic influences experienced by Malagasy since the initial colonization of the Island.

Another open problem concerns the pre-Indonesian ancestral population. It is still debated whether the island was inhabited before the Indonesian colonization. In case the answer is positive it may be possible to track the aboriginal vocabulary in the dialects. For example, the Mikea are the only hunter-gatherers in Madagascar, and it is unclear whether they are a relic of the aboriginal pre-Indonesian population or just ‘ordinary’ Malagasy who switched to a simpler economy for historical reasons. If the first hypothesis is the correct one, they should show some residual aboriginal vocabulary in their dialect, and the same is expected for the neighboring populations of Vezo and Masikoro.

## Methods

The method that we have used is based on an automated measure of lexical distance between pairs of words with same meaning contained in Swadesh lists [9], [10] .

The use of Swadesh lists [7] in lexicostatistics has been popular for half a century. These are lists of words associated with the same 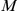 meanings (the original Swadesh choice was 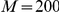), which refer to the basic activities of humans. Comparing the two lists corresponding to a pair of languages it is possible to determine the percentage of shared *cognates*, which is related to their lexical distance. Recent examples of the use of Swadesh lists and cognate counting to construct language trees are the studies of Gray and Atkinson [12] and Gray and Jordan [13].

The idea of measuring relationships among languages using vocabulary is much older than lexicostatistics and it seems to have its roots in the work of the French explorer Dumont D'Urville. He collected comparative word lists during his voyages aboard the Astrolabe from 1826 to 1829 and, in his work about the geographical division of the Pacific [14], he proposed a method to measure the degree of relation among languages. He used a core vocabulary of 115 terms, then he assigned a distance from 0 to 1 to any pair of words with the same meaning and finally he was able to determine the degree of relation between any pair of languages.

Our automated method works as follows: for any language we write down a Swadesh list, then we measure the distance between words with the same meaning belonging to different languages and, finally, we obtain the distance between two languages by an average. This approach is motivated by the analogy with genetics: vocabulary has the role of DNA and the comparison is simply made by measuring the differences between the DNA of the two languages. There are various advantages: the first is that, at variance with previous methods, it avoids subjectivity; the second is that results can be replicated by other scholars assuming that the database is the same; the third is that it does not require specific expertise in linguistics; and the last, but surely not the least, is that it allows for a rapid comparison of a very large number of languages (or dialects).

More precisely, our measure of lexical distance between two words is a variant of the Levenshtein distance [15]. The Levenshtein distance is simply the minimum number of insertions, deletions, or substitutions of a single character needed to transform one word into the other. Our distance is obtained by a normalization.

Given two words 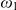 and 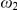, our measure of distance 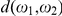 is
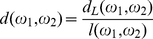
(1)where 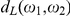 is their standard Levenshtein distance and 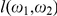 is the number of characters in the longer of the two words 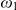 and 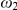. Therefore, the distance can take any value between 0 and 1.

The reason for the normalization can be understood by the following example. Consider the case of two words of the same length in which a single substitution transforms one word into the other. If they are short, let's say 2 characters, they are very different. On the contrary, if they are long, let's say 8 characters, it is reasonable to say that they are very similar. Without normalization, their distance would be the same, equal to 1, regardless of their length. Instead, introducing the normalization factor, in the first case the distance is 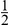, whereas in the second, it is much smaller and equal to 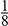.

We use distance between pairs of words, as defined above, to construct the lexical distances between languages. For any language we prepare a list of words associated with the same 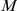 meanings (we adopt the original Swadesh choice of 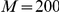).

Assume that the number of languages is 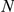 and any language in the group is labeled by a Greek letter (say 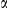) and any word of that language by 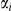 with 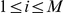. The same index 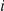 corresponds to the same meaning in all languages, i.e., two words 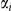 and 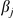 in the languages 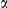 and 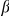 have the same meaning if 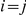.

The lexical distance between two languages is then defined as
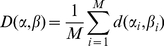
(2)It can be seen that 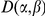 is always in the interval [0,1] and obviously 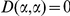.

The result of the analysis described above is a 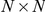 upper triangular matrix whose entries are the 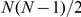 non-trivial lexical distances 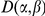 between all pairs of languages.

If a family of languages is considered, all the information about their relationships is encoded in the associated matrix, but this information is not manifest and it has to be extracted. The ubiquitous approach to this problem is to transform the matrix information into a phylogenetic tree.

Nevertheless, in this transformation, part of the information may be lost because transfer among languages is not exclusively vertical (as in mtDNA transmission from mother to child) but it also can be horizontal (borrowings and, in extreme cases, creolization). In previous papers we proposed a complementary geometric approach [16], [5] (Structural Component Analysis). This approach encodes the matrix information into the positions of the languages in a 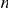-dimensional space. For large 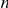 one recovers all the matrix content, but a low dimensionality, typically 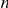 = 2 or 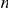 = 3, is sufficient to grasp all the relevant information. However, results shown in the last two figures of this paper mostly rely on a direct investigation of the entries of the matrix and to simple averages over them.

## Supporting Information

Table S1 This table provides information on the people who furnished the data collected by the author at the beginning of 2010. For any dialect two consultants have been independently interviewed. Their names and birth dates follow each of the dialect names.
(PDF)

Table S2This table contains the 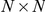 upper triangular matrix which is the result of the analysis described in the Methods section of the paper. The entries of the matrix are the 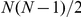 non-trivial lexical distances 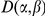 between all pairs of languages.(PDF)
