# Supplementary material for: The Settlement of Madagascar: What Dialects and Languages Can Tell Us
Source: PLoS One. 2012 Feb 21;7(2):e30666. doi: 10.1371/journal.pone.0030666 (PMC3283610; doi:10.1371/journal.pone.0030666)
Supplement: Table S1 — This table provides information on the people who furnished the data collected by the author at the beginning of 2010. For any dialect two consultants have been independently interviewed. Their names and birth dates follow each of the dialect names. (PDF) [file pone.0030666.s001.pdf]

Table 1: People which furnished the data on Malagasy dialects

**MERINA**		
**(ANTANANARIVO)**	SERVA Maurizio	
**ANTANOSY**	SOAFARA Joselina Nere	08 November 1987
**(TOLAGNARO)**	ETONO Imasinoro Lucia	18 February 1982
**BETSIMISARAKA**	ANDREA Chanchette Généviane	07 August 1985
**(FENOARIVO-EST)**	RAZAKAMAHEFA Joachim Julien	09 November 1977
**SAKALAVA**	SEBASTIEN Doret	26 November 1980
**(MORONDAVA)**	RATSIMANAVAKY Christelle J.	29 February 1984
**VEZO**	RAKOTONDRABE Justin	02 August 1972
**(TOLIARA)**	RASOAVAVATIANA Claudia S.	28 June 1983
**ZAFISIRO**	RALAMBO Alison	11 June 1982
**(FARAFANGANA)**	RAZANAMALALA Jeanine	03 February 1980
**ANTAIMORO**	RAZAFENDRALAMBO Haingotiana	24 July 1985
**(MANAKARA)**	RANDRIAMITSANGANA Blaise	05 February 1989
**ANTAISAKA**	RAMAHATOKITSARA Fidel Justin	24 April 1984
**(VANGAINDRANO)**	FARATIANA Marie Luise	17 August 1990
**ANTAMBOHOAKA**	RAKOTOMANANA Roger	04 May 1979
**(MANANJARY)**	ZAFISOA Raly	20 April 1983
**BETSILEO**	RAMAMONJISOA Andrininina Leon Fidelis	16 April 1987
**(FIANARANTSOA)**	RAKOTOZAFY Teza	25 December 1985
**BARA**	RANDRIANTENAINA Hery Oskar Jean	17 Jenuary 1986
**(BETROKA)**	NATHANOEL Fife Luther	26 May 1983
**TSIMIHETY**	RAEZAKA Francis	23 December 1984
**(MANDRITSARA)**	FRANCINE Germaine Sylvia	04 May 1985
**MAHAFALY**	VELONJARA Larissa	21 April 1989
**(AMPANIHY)**	NOMENDRAZAKA Christian	07 June 1982
**SIHANAKA**	ARINAIVO Robert Andry	06 Jenuary 1979
**(AMBATONDRAZAKA)**	RONDRONIAINA Natacha	27 December 1985
**ANTANKARANA**	ANDRIANANTENAINA N. Benoit	06 August 1984
**(VOHEMAR)**	EDVINA Paulette	28 Janurary 1982
**ANTANKARANA**	RANDRIANARIVELO Jean Ives	24 December 1986
**(ANTALAHA)**	RAZANAMIHARY Saia	07 September 1985
**SAKALAVA**	CASIMIR Jaozara Pacific	03 April 1983
**(AMBANJA)**	ZAKAVOLA M. Sandra	17 July 1984
**SAKALAVA**	RATSIMBAZAFY Serge	17 May 1978
**(MAJUNGA)**	VAVINIRINA Fideline	23 June 1970
**ANTANDROY**	RASAMIMANANA Z. Epaminodas	05 June 1983
**(AMBOVOMBE)**	MALALATAHINA Tiaray Samiarivola	07 July 1984
**MASIKORO**	MAHATSANGA Fitahia	22 March 1976
**(ANTALAHA)**	VOANGHY Sidonie Antoinnette	12 October 1981
**ANTANKARANA**	BAOHITA Maianne	21 August 1984
**(AMBILOBE)**	NOMENJANA HARY Jean Pierre Felix	07 June 1980
**SAKALAVA**	HANTASOA Marie Edvige	02 November 1985
**(MAINTIRANO)**	KOTOVAO Bernard	06 October 1983
**BETSIMISARAKA**	RASOLONANDRASANA Voahirana	24 September 1985
**(MAHANORO)**	ANDRIANANDRASANA Maurice	03 April 1979
