# Supplementary material for: The Settlement of Madagascar: What Dialects and Languages Can Tell Us
Source: PLoS One. 2012 Feb 21;7(2):e30666. doi: 10.1371/journal.pone.0030666 (PMC3283610; doi:10.1371/journal.pone.0030666)
Supplement: Table S2 — This table contains the upper triangular matrix which is the result of the analysis described in the Methods section of the paper. The entries of the matrix are the non-trivial lexical distances between all pairs of languages. (PDF) [file pone.0030666.s002.pdf]

Table 2. The matrix of the lexical distances of the 23 Malagasy dialects, with entries multiplied by 1000, is the following:

1																						
2	323																					
3	246	276																				
4	322	240	295																			
5	302	281	309	345																		
6	227	318	275	359	266																	
7	413	386	390	418	314	370																
8	280	386	342	401	356	245	436															
9	366	424	379	412	405	375	450	409														
10	411	396	416	440	318	366	249	456	482													
11	207	326	260	362	286	061	383	201	374	384												
12	362	343	345	387	292	328	289	397	435	330	324											
13	303	369	330	381	384	329	454	362	256	487	318	407										
14	343	302	331	355	243	317	303	403	423	314	336	301	419									
15	397	453	394	462	392	375	342	463	485	304	383	405	471	388								
16	368	391	385	416	392	390	448	406	320	474	383	429	325	418	486							
17	400	350	369	390	280	358	165	433	427	278	373	240	439	261	358	410						
18	322	376	325	374	391	337	426	381	198	473	339	412	234	406	461	264	414					
19	358	407	376	417	408	394	440	419	292	481	387	431	325	422	472	161	408	243				
20	297	388	359	430	356	299	400	346	386	433	275	375	363	375	455	348	394	349	355			
21	386	341	370	385	290	344	262	403	422	321	348	250	404	306	403	401	213	416	417	383		
22	225	389	332	394	382	316	471	319	385	475	287	421	296	431	480	382	467	348	387	356	441	
23	379	424	407	424	398	380	443	433	315	466	380	412	351	420	472	203	395	288	202	351	409	406
	1	2	3	4	5	6	7	8	9	10	11	12	13	14	15	16	17	18	19	20	21	22

where the number-variant correspondence is:
1 Antambohoaka (Mananjary), 2 Antaisaka (Vangaindrano), 3 Antaimoro (Manakara), 4 Zafisoro (Farafangana), 5 Bara (Betroka), 6 Betsileo (Fianarantsoa), 7 Vezo (Toliara), 8 Sihanaka (Ambatondranzaka), 9 Tsimihety (Mandritsara), 10 Mahafaly (Ampanihy), 11 Merina (Antananarivo), 12 Sakalava (Morondava), 13 Betsimisaraka (Fenoarivo-Est), 14 Antanosy (Tolagnaro), 15 Antandroy (Ambovombe), 16 Antankarana (Vohemar), 17 Masikoro (Miary), 18 Antankarana (Antalaha), 19 Sakalava (Ambanja), 20 Sakalava (Majunga), 21 Sakalava (Maintirano), 22 Betsimisaraka (Mahanoro), 23 Antankarana (Ambilobe).
